# Characterization and Cellular Toxicity Studies of Commercial Manganese Oxide Nanoparticles

**DOI:** 10.3390/nano14020198

**Published:** 2024-01-16

**Authors:** Linda J. Johnston, Xiaomei Du, Andre Zborowski, David C. Kennedy

**Affiliations:** 1 Metrology, National Research Council Canada, 1200 Montreal Road, Ottawa, ON K1A 0R6, Canada; linda.johnston@nrc-cnrc.gc.ca; 2 Energy, Mining and Environment, National Research Council Canada, 1200 Montreal Road, Ottawa, ON K1A 0R6, Canada; xiaomei.du@nrc-cnrc.gc.ca (X.D.); andre.zborowski@nrc-cnrc.gc.ca (A.Z.)

**Keywords:** nanoparticles, cytotoxicity, reactive oxygen species, transmission electron microscopy, dynamic light scattering

## Abstract

Manganese oxide nanoparticles (MnO_x_ NPs) are finding applications in several environmentally important areas such as farming and energy storage. MnO_x_ NPs span a range of metal oxidation states that open up a wide range of applications in catalysis as well. As a result, it is important to understand how such materials can impact human health through incidental exposure. In this study, we examined a range of commercially available Mn_2_O_3_ NPs and compared our characterization data to those supplied by manufacturers. Discrepancies were noted and then measured values were used to assess the biological impact of these materials on three mammalian cell lines—A549, HepG2 and J774A.1 cells. Cell toxicity assays showed that all Mn_2_O_3_ particles exhibited cytotoxic effects that may be correlated, at least in part, to the production of reactive oxygen species. All eight nanoforms also activated caspase 3 but not caspase 1, although the magnitude of these changes varied greatly between materials.

## 1. Introduction

Manganese oxide nanoparticles (MnOx NPs) are used in a range of applications, including energy storage, catalysis, soil remediation and sustainable agriculture [1,2]. The relatively low cost, high abundance and anticipated low toxicity of manganese and the presence of multiple manganese oxidation states which facilitate a range of redox processes contribute to their widespread use. Manganese is an essential element, being a cofactor needed for the activity of several enzymes, and is important in controlling cellular redox status, urea metabolism and neurotransmitter function. However, exposure to elevated levels of manganese, for example in industrial settings, is a significant occupational risk factor and has been suggested to lead to neurological problems.

A variety of MnO_x_ NPs (MnO, MnO_2_, Mn_2_O_3_ and the mixed valence (II/III) Mn_3_O_4_) are used for biomedical applications [1], as summarized in recent reviews [3]. The mode of action and the overall reactivity vary with the manganese oxidation state, particle size, surface chemistry and the cellular environment. One important application area is the development of new magnetic resonance imaging (MRI) contrast agents (typically using MnO- or Mn_3_O_4_-based materials) that overcome some of the issues associated with the commonly used gadolinium complexes and potentially improve efficacy [4]. Other application areas include uses as anti-parasitic agents (Mn_2_O_3_) [5] and anti-cancer therapeutics (Mn_2_O_3_) [6], treatment of bacterial infection [7] and the development of theranostic agents that can be used for combined MRI diagnostics and targeted drug delivery. The range of potential applications and the complexity of the chemistry of manganese oxides make it essential to carry out detailed studies of their potential deleterious biological impact. Such studies have been hampered to some extent by the range of manganese oxides that are used and the complexity of their chemistry. As an example, MnO_x_ NPs have been shown to exhibit enzyme-like activity that can either reduce or increase oxidative stress. A recent study examined the competition between the pro- and anti-oxidant behavior for MnO_2_, Mn_2_O_3_ and Mn_3_O_4_ by studying their catalytic activity towards ascorbate and glutathione antioxidants and reactive oxygen species [8]. The three NPs showed different reactivity towards the various substrates and the reactions with ascorbate were shown to consume oxygen and lead to formation of ascorbyl radicals. Furthermore, cellular assays demonstrated either a decrease in cell viability or a reduction in peroxide mediated cytotoxicity depending on conditions. In other studies, oxidative stress induced by MnO_x_ NPs has been shown to lead to cell death via a caspase 3 pathway [9,10]. In contrast, the ability of Mn_3_O_4_ to mimic the behavior of multiple enzymes that are involved in regulating oxidative stress has been suggested to provide a potential candidate for therapeutic applications for oxidative stress-induced neurological disorders such as Parkinson’s disease [11]. Both cytotoxicity and an antioxidant effect have been observed for mixed phase manganese oxide NPs, highlighting the interplay of factors that influence the biological outcome [12]. NP dissolution is also an important factor in determining the potential biological impact of MnO_x_ [13]. Dissolution of MnO_x_ NPs to give Mn^2+^ is a mechanism for enhanced sensitivity for some MRI applications [14]. Nevertheless, the dissolution of MnO_x_, particularly in an acidic environment such as found in lysosomal fluids, may in other cases be responsible for deleterious effects on cells.

This complexity makes it impossible to draw general conclusions on potential toxicity across MnO_x_ nanomaterials and necessitates a careful assessment of potential biological impact for materials that will be used in either consumer goods or biomedical applications. The biological studies must also be correlated with detailed physical–chemical characterization, as has been noted in a number of previous studies of the biological effects of metal oxide NPs [15,16,17]. For example, a detailed study of cytotoxicity for a series of eight metal oxide NPs demonstrated that surface charge, available binding sites and ion dissolution all play an important role in determining cytotoxicity [18]. Related work confirmed that both cell apoptosis and suppression of cell proliferation [19] contribute to the observed cytotoxicity. Herein, we report toxicity data for eight commercial Mn_2_O_3_ NPs in order to assess differences and similarities between materials available for purchase. We selected samples from different suppliers and with different reported sizes and surface coatings in order to determine whether trends in physical properties and cytotoxicity could be observed. Physical characterization data for all samples were obtained using a combination of transmission electron microscopy (TEM) for measuring the primary particle size, specific surface area (SSA) to determine the available surface area of the particles, dynamic light scattering (DLS) to measure the hydrodynamic diameter (size) when suspended in water and zeta potential measurements to measure the surface charge, while sample purity was determined by inductively coupled plasma mass spectroscopy (ICP-MS). A DLS study of the stability of the NP dispersion in the cell culture medium was also performed to determine if aggregation or agglomeration was occurring over the time course of the cell-based assays. Cellular assays were performed in three cell lines—A549 human lung epithelial cells (A549), HepG2 human hepatocyte cells (HepG2) and J774A.1 mouse macrophage cells (J774A.1). These cell lines were selected because they are often used in other studies and thus form a point for interstudy comparison. Additionally, A549 cells are a common cell line used to study inhalation exposure, while HepG2 cells are used as the liver is a common endpoint for the localization of many nanomaterials in vivo. J774A.1 cells were selected as they typically show increased susceptibility to nanoparticle toxicity as they take particles in more efficiently than other cell lines and thus can give information as to potential concern when other cells do not show any effects. Two orthogonal cytotoxicity assays were performed—the MTT assay at 24, 48 and 72 h and the Neutral Red Assay at 72 h. A DCFDA assay for reactive oxygen species (ROS) was performed at 24 h, as were assays to determine caspase 1 and caspase 3 activity.

## 2. Materials and Methods

**Materials.** Mn_2_O_3_ NPs were purchased from US Research Nanomaterials (Mn-01, Mn-02, Mn-03, Mn-04, Mn-05), mKNano (Mn-06, 50 nm), American Elements (Mn-07, <100 nm) and Nanografi (Mn-08, 28 nm). The sample code, supplier, surface coating and nominal particle size provided by the supplier are listed in Table 1.

**Specific surface area.** SSA was measured on an ASAP 2020 system (Micromeritics, Norcross, GA, USA) using the Brunauer Emmett Teller (BET) method with nitrogen adsorption. The samples were stored in an oven at 60 °C before measurements and the sample cell was weighed before degassing. Samples were heated at 10 °C/min to 110 °C. They were then held for 10 min at this temperature before being heated to 200 °C at 10 °C/min and held for an additional 2 h prior to analysis of SSA at 77 K. Specific surface area was determined by the multipoint BET method with a minimum of 5 points taken between relative partial pressure values from 0.05 to 0.3 where the adsorption curve is linear.

**Transmission electron microscopy.** Aqueous dispersions (prepared as outlined below) were deposited on plasma treated (30 sccm 75%/25% Ar/O_2_, 2 min, ≈40 W using a Fischione 1070, NanoClean, Export, PA, USA) carbon film covered copper grids (200 mesh, Ted Pella 01840-F, Redding, CA, USA). 10 μL of 0.1 mg/mL metal oxide suspension was added to the carbon film, and the water was wicked away after 10 min using a piece of filter paper. The samples were then immersed in deionized water twice and allowed to dry, typically for 1–2 h. Additional rinse steps and dispersion volumes were tested for some samples in attempts to reduce the level of agglomeration, although without substantial improvement. TEM images were recorded on a Titan3 80–300 FEI microscope operated at 300 kV and calibrated with a TEM magnification standard (MAG*I*CAL, EMS). Images were analyzed with ImageJ ver. 1.51a using the polygon outlining feature to trace individual particles and record their area, perimeter, Feret and minFeret. Area was converted to an equivalent circular diameter and the aspect ratio was calculated as the Feret/minFeret ratio. Particle size histograms and smooth kernel probability distributions were plotted and statistics were calculated in OriginPro 2019.

**Dispersion of nanoparticles**. All NPs were suspended in water at 1 mg/mL using probe sonication. Samples were sonicated with a 130 W ultrasonic processor (EW-04714-50, Cole-Parmer, Quebec City, QC, Canada) equipped with a 1/4 inch tip probe (EW-04712-14 Cole-Parmer) for 30 min with a 30 s on/off pulse cycle with a 50% amplitude. The initial suspensions were diluted in water for DLS, zeta potential and TEM experiments. For cell experiments, NP suspensions were prepared by mixing the stock aqueous suspension with an equivalent amount of complete DMEM medium with 10% FBS and 1% pen/strep. This diluted the stock by 50% and resulted in suspensions of equal parts water and medium. Subsequent dilutions of this stock were made using a medium diluted 50% with water to maintain the same serum concentration in the medium at all concentrations. Afterward, 100 µL of diluted nanoparticle suspensions was added to wells that had been seeded with 100 µL cells in medium the previous day. This serum concentration was also used for DLS experiments in the cell culture medium.

**Dynamic light scattering and zeta potential measurements**. Samples were run on a Malvern (Westborough, MA, USA) Zetasizer Nano-ZS (632.8 nm HeNe laser and signal detection at 173°). Samples in water were measured in a zeta potential cuvette for both DLS and zeta potential measurements at 100 µg/mL. Samples for DLS measurements in the cell culture medium were run in plastic cuvettes (BRAND) with a 1 mL sample volume. Size measurements (equivalent hydrodynamic diameter, Z-average and polydispersity index, PDI) were taken at 25 °C. Samples were equilibrated for 180 s before starting the measurements and 3 runs, each consisting of ten measurements of 10 s, were acquired. DLS measurements were analyzed using Zetasizer software (ver. 7.11) via the cumulants method with the general-purpose model. The data were processed to obtain a three-measurement average value for each sample with corresponding standard deviations for Z-average and PDI. The pH of the samples was not adjusted upon suspension in water. All particle suspensions were between pH 6 and 7 in water at 100 µg/mL. While adjusting the pH can change the charge on the particle surface and improve the stability of the suspension in water, neutral suspensions are needed for performing cell experiments and adjusting the pH to control the zeta potential can overpower the buffering capacity of the cell culture medium. The NPs were suspended in water and diluted with the cell culture medium as described above. Afterward, 500 µL of the sample was mixed with the same amount of medium to achieve a 7.5% serum concentration consistent with that used in the cell culture experiments. Subsequent dilution was performed using a 75% cell culture medium and 25% water solution in order to dilute cells to 100 µg/mL for DLS without adjusting the serum concentration. Samples were measured immediately upon preparation and then every 24 h for 3 days. In between measurements, samples were incubated in the cell incubator at 37 °C. Each day samples were resuspended before measuring as the particles had all settled out of suspension.

**Cell Culture.** A549, HepG2 and J774A.1 cells (American Tissue Culture Center, Cedarlane, Burlington, ON, Canada) were grown in Dulbecco’s modified Eagle’s medium (DMEM) (Gibco) containing 10% fetal bovine serum (FBS) (Gibco) and 1% penicillin–streptomycin (Pen/strep) (50 µg/mL, Gibco) under standard culture conditions (37 °C, 5% CO_2_). T75 flasks (Falcon) were used to maintain the cells and Trypsin-EDTA solution (Gibco) was used for passaging the A549 and HepG2 cells (2 mL per T75 flask). A cell scraper was used to detach J774A.1 cells from the flask and an aliquot was then removed and passaged into a new flask every 3–4 days.

**MTT Assay.** Cells were seeded into wells in a 96-well plate (Falcon, ThermoFisher Scientific, Markham, ON, Canada) (1 × 10^5^ cells/mL, 100 µL per well) to cover an 8x6 grid, filling 48 wells. The remaining wells were filled with 200 µL of water. Diluted samples were prepared spanning from 500 µg/mL to 5 µg/mL in a 1:1 mixture of water and cell medium. After 24 h, 100 µL volumes of these diluted samples were added to the corresponding wells containing 100 µL of medium and cells already, resulting in final concentrations of 250 µg/mL to 2.5 µg/mL. Samples were diluted across this range as described above to ensure that the serum concentration remained constant. For each sample, three replicates were performed as well as three replicates of an untreated control containing just water and medium but no NPs. Each plate was set up in triplicate to take measurements over 3 days. Cells were then incubated with NPs for 24, 48 or 72 h. On each day, one plate was removed and the workup consisted of adding a 50 µL PBS solution of MTT (2.5 mg/mL) to each well. The plate was then returned to the incubator for 3 h. After 3 h, the plates were removed from the incubator and all the solution in the wells was aspirated, leaving purple formazan crystals in those wells with viable cells. Afterward, 150 µL of DMSO was added to each well and plates were agitated manually to dissolve the crystals; 100 µL of the resulting solutions in each well was transferred to a fresh plate in order to remove scattering from precipitated particles that affected the readings. The absorbance at 570 nm was measured in each well using a plate reader (Fluorstar Omega, BMG Labtech, Ortenburg, Germany). IC_50_ values were determined by plotting the absorbance at each concentration compared to that of the untreated control (100% viability) for each particle in each cell line at each time point. Three replicates were performed for each sample on each cell line at each time point.

**Neutral Red Assay.** This procedure was modified from a published protocol [20]. First, 96-well plates with cells were prepared in the same manner as described for the MTT assay. This assay; however, was only performed on cells incubated for 72 h, so only one replicate of each plate was prepared. Neutral red medium was prepared as reported [20]. After 72 h, the plates were removed from the incubator and the cell medium was aspirated. Cells were then washed once with 150 µL of PBS before adding 100 µL of neutral red medium to each well. The plates were incubated for 2 h with the neutral red dye. After 2 h, the plates were removed from the incubator and the medium was aspirated. Each well was then washed with 150 µL of PBS, before adding 150 µL of destain solution (50% ethanol, 49% water, 1% acetic acid) to each well. Plates were agitated manually on the bench to dissolve the dye. Then, 100 µL of solution from each well was transferred to a new plate to remove interferences from precipitated NPs on the bottom of the wells. The absorbance of each well was measured at 540 nm and the results were plotted in the same manner as for the MTT assay.

**DCFDA Assay.** Cells were plated into 96-well plates with clear bottoms and black walls in the same manner as described above for the MTT assay and incubated for 24 h. DCFDA buffer and solution were prepared as per the assay kit protocol (Abcam, Cambridge, UK) immediately before use. After 24 h, the cell medium was aspirated and the wells were washed with 100 µL DCFDA buffer. Afterward, 100 µL DCFDA solution was added to each well and the plates were incubated for 45 min at 37 °C. After incubation, the DCFDA solution was removed from the wells and 100 µL 1X PBS was added to each well. The fluorescence was measured at Ex/Em 485/535 using a spectrophotometer. The PBS was then aspirated and 100 µL of cell culture medium was added to each well. Dilutions of NPs, prepared as described above, were then added in a manner identical to the MTT assay, and plates were scanned immediately to measure the baseline fluorescence in each well. Fluorescence measurements were taken again at Ex/Em = 485/535 nm after 1, 2, 3 and 4 h. Little effect was observed for all samples, so they were then incubated overnight and the fluorescence intensity measured again at 24 h. Between each time at which the fluorescence was measured, the plates were incubated under standard culture conditions.

**Caspase 1 and 3 assays**. The caspase 1 and caspase 3 assays were performed using luminescent kits purchased from Promega (Madison, WI, USA). Both assays were performed as recommended in the detailed procedure associated with the kits. Cells were seeded for 24 h before the addition of the NPs for an additional 24 h. Cells were then rinsed and incubated with the appropriate dyes for 3 h at room temperature in the dark according to the kit protocols (results from a 1 h incubation were less consistent).

**ICP-MS.** The samples were measured using an ICP-MS triple quadrupole 8800 (Agilent, Santa Clara, CA, USA). Sample digestion, measurement and analysis were performed by the University of Ottawa Geochemistry Laboratory core facility staff.

## 3. Results and Discussion

### 3.1. Nanomaterial Characterization

**Figure 1:**
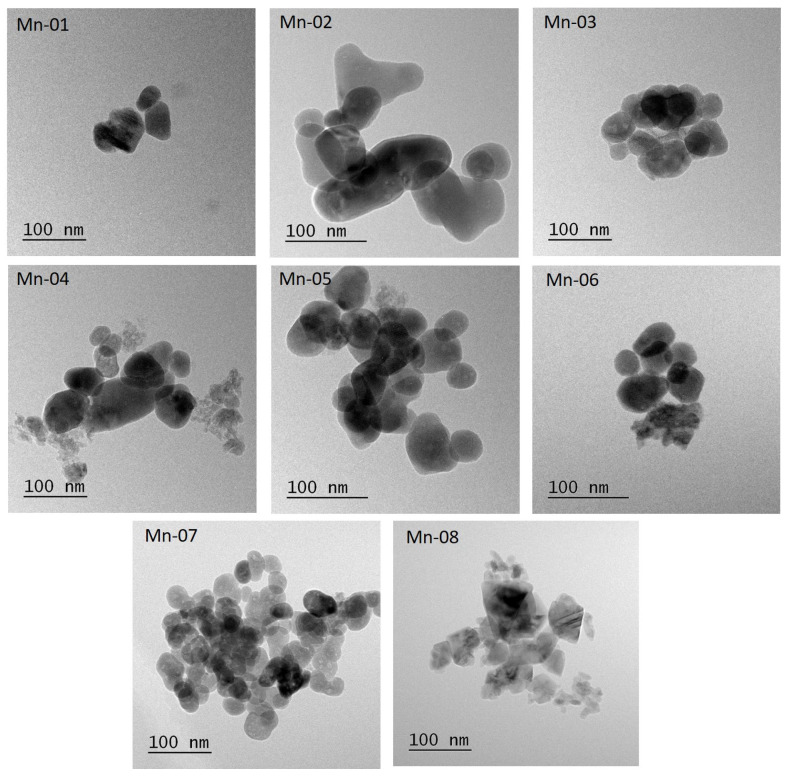
Representative TEM images for Mn_2_O_3_ NPs.

Particle size and morphology were assessed by TEM with samples deposited on carbon film grids from an aqueous dispersion. Representative images are shown in Figure 1; these images are selected to show areas with some analyzable individual particles. Additional images at lower magnification (Figure S1) illustrate the variation in sample morphology that is observed for the various samples. Overall, the images indicate that there was a significant level of aggregation/agglomeration for each sample, similar to observations for a number of other metal oxide NPs [21]. Several variations of the sample deposition method were tested but failed to provide a significant reduction in the heterogeneity for the deposited samples. The images show a range of particle shapes (circular, elliptical, irregular and rectangular or square usually with rounded corners) and contrast, as summarized in Table 2. In addition, some areas had lower contrast rectangular or irregularly shaped features that appear to be plates or rods (Figure S1, Mn-04, Mn-06, Mn-07); note that these features were typically overlapped with other particles and therefore not included in the size analysis summarized below. Note that this conclusion is consistent with the shape data provided by the supplier (Table 2). There are also areas with indistinct features that may be relatively small particles (Figure 1, Mn-04; Figure S1, Mn-01, Mn-05, Mn-06).

**Figure 2:**
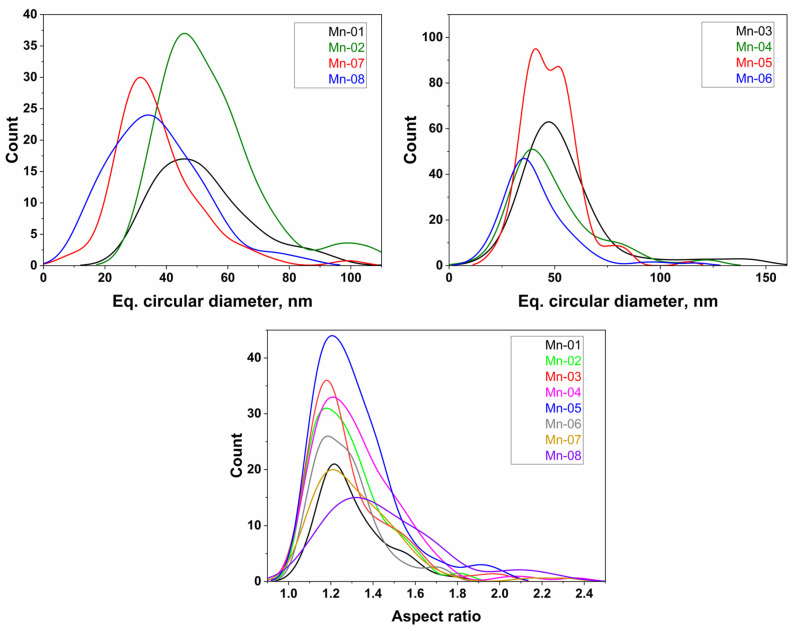
Kernel-smoothed histograms for equivalent circular diameter and aspect ratio for all Mn_2_O_3_ NPs.

Particles were analyzed as summarized in the Materials and Methods Section and the data are summarized in Table 2, along with size information provided by the supplier. The number of particles analyzed, mean equivalent diameter (and standard error), standard deviation (SD, as a measure of the breadth of the particle size distribution) and aspect ratio and SD are provided for each sample. Particle agglomeration made the size analysis particularly challenging for some samples (Mn-01, Mn-06, Mn-07 and Mn-08) and fewer particles were analyzed in these cases. Histograms for equivalent diameter are shown for each sample in Supporting Information, Figures S2 and S3. The probability density plots for both equivalent diameter and aspect ratio for each sample are shown in Figure 2. The latter provides a quick visual comparison of data sets. It is important to consider that a very small fraction of particles in most images is analyzed. In some cases, there are large “features” that are not counted either because they overlap other particles or their edges are ill-defined. In other cases, there are very small poorly defined particles that are also not counted. These complications as well as the small number of particles analyzed (estimated to be <5% of the total) may lead to inaccuracy in the reported particle size distributions.

The mean equivalent diameter for the eight samples ranged from 37 to 56 nm (Table 2), covering a relatively modest range of sizes and the distribution widths are relatively broad (>15 nm). Note that in most cases the values were somewhat higher than the nominal size provided by the supplier. Despite a diversity of shapes, the aspect ratios were 1.3, with one exception (Mn-08, 1.5). Note that the aspect ratio does not include the rod-shaped particles or plates that were detected for several samples but were always overlapping with other particles, although these account for a small number of particles in the images.

Specific surface areas were measured by BET for all Mn_2_O_3_ samples and are summarized in Table 1, along with the nominal values provided by the supplier where available. The measured SSA values were similar to the supplier data for Mn-07 and Mn-05. The measured values were all ~35 m^2^/g or lower for each of the USRN 30 nm NPs and for Mn-08; these values are much lower than the values of 150 m^2^/g and >155 m^2^/g provided by the supplier for Mn-01 and Mn-08. Overall, these values are consistent with aggregated or agglomerated samples. The agreement between measured SSA and manufacturer data was poor for more than half of the samples. It should be noted that the decrease in SSA for PVP and stearic-acid-modified NPs is consistent with our earlier results for both NiO and CeO_2_ NPs [22,23]. In both cases, the measured SSA decreased slightly for the PVP-coated sample compared to the bare sample of the same size from the same supplier; there was a considerably larger decrease for samples containing stearic acid, which may indicate that the addition of a long chain fatty acid introduces issues with measurement of SSA. Data on SSA are either not reported by the supplier or are provided as a relatively broad range for both PVP and stearic-acid-modified NiO, CeO_2_ and Mn_2_O_3_ NPs.

The particle size for aqueous dispersions of Mn_2_O_3_ NPs was assessed by DLS and the results are summarized in Table 3, along with zeta potential measurements for each sample. The particle sizes, as measured by the hydrodynamic diameter, Z-average, ranged from 200 to 270 nm for seven of the samples, with polydispersities (PDI) from 0.24 to 0.36. This indicates that the particle size in aqueous dispersion is considerably larger than the TEM diameter (or the nominal size provided by the supplier) measured for individual particles. The results also suggest that the agglomeration problems encountered with the TEM samples are likely to be primarily due to issues with sample deposition on the TEM grid. The stearic-acid-coated sample (Mn-03) did not disperse well in water and had a large size and very high polydispersity. Zeta potentials were positive for all samples except the PVP-coated Mn-02, which had a negative zeta potential.

ICP-MS analysis was also performed to determine the purity of the Mn oxide samples and the percentage of Mn in each sample (Table 3). All samples had a Mn content below the theoretical value of 69.6% for Mn_2_O_3_. Only Mn-08 showed significant metallic impurities (Cu 0.4%). There was considerable variation between subsequent digestions, suggesting that the low values for Mn could be the result of incomplete digestion. Alternatively, the presence of organic impurities could result in a lower Mn measurement.

DLS experiments for dispersions prepared in cell culture medium were measured over a period of 3 days and the results are summarized in Table 4. The Z-average and PDI measured in the cell culture medium were very similar to the data obtained in water and the measured values were reasonably stable over a period of 72 h, which is the maximum time required for the biological assays. These results are in contrast to those obtained in our earlier work on other metal oxide NPs for which the hydrodynamic diameter and polydispersity typically increased in cell culture medium compared to water and continued to increase with storage time [22,23].

### 3.2. Cell Assays

There is relatively little scientific literature on the cellular toxicity of Mn_2_O_3_ nanoparticles compared to other forms of manganese oxide. The available literature arrives at very different conclusions about the safety of such particles with some studies reporting very little cytotoxicity and others reporting clear dose-dependent effects [1,5,6,19]. This, of course, can arise when using different specific materials, in different cell lines, with different assays and in different cell culture medium compositions. All of these factors can impact the results. To try to resolve some of the issues, we performed two different assays to assess cellular cytotoxicity in three cell lines: A549 (human lung), HepG2 (human liver) and J774A.1 (mouse macrophage) for each of the eight nanoforms of Mn_2_O_3_ employed in this study. Cytotoxicity was measured at 24 h, 48 h and 72 h using an MTT assay and results were then corroborated at 72 h using a neutral red assay. In all three cell lines, an increase in cytotoxicity was observed from 24 h to 48 h, while the 48 h and 72 h cytotoxicity curves were generally very similar. Furthermore, a DCFDA assay was performed to determine the effect of the particles on oxidative stress and, for the macrophages, caspase 1 and caspase 3 activation assays were performed to determine if the particles were inducing an immune/inflammatory response.

### 3.3. A549 Lung Cells

**Figure 3:**
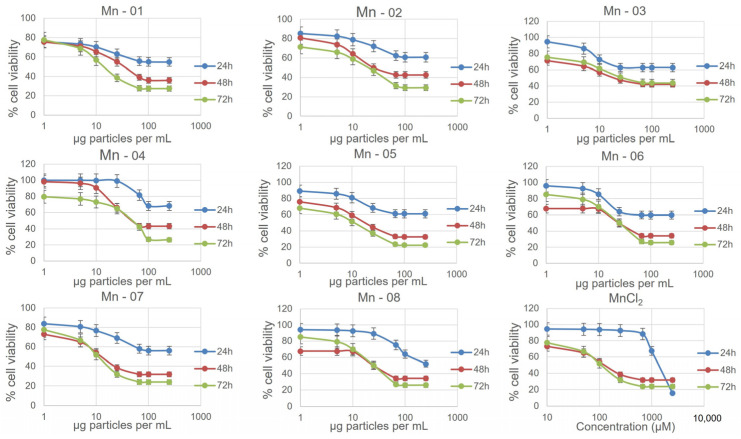
Nanoparticle cytotoxicity in A549 cells measured using the MTT assay at 24, 38 and 72 h.

**Figure 4:**
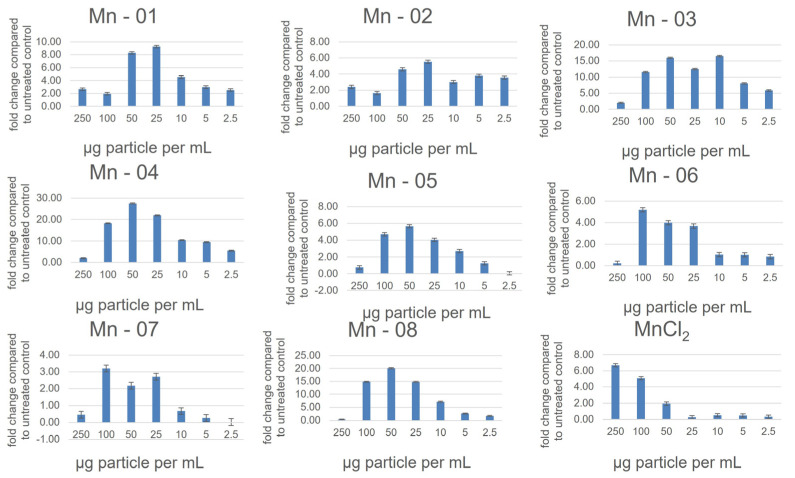
Reactive oxygen species as measured using a DCFDA assay in A549 cells after 24 h of exposure.

In A549 cells (Figure 3), there was little difference between the IC_50_ values and cytotoxicity curves of all eight nanoforms. There was some variation observed for the neutral red assay, though this assay notably showed less cytotoxicity compared to the MTT assay, with cell viability at 72 h ranging from 60–90 percent at 250 µg/mL (Figure S4). In this assay, Mn-02 and Mn-03 showed the least cytotoxicity, while the shape of the toxicity curve for Mn-04 differed from other samples. It was the least cytotoxic until approximately 75 µg/mL, above which concentration the cell viability dropped more quickly compared to other samples. This is interesting as these three samples are the only coated samples, suggesting that bare particles are more cytotoxic than coated particles with the nature of the coating having a significant impact on particle stability but little impact on cytotoxicity. Tolliver et al. reported the cytotoxicity of a single Mn_2_O_3_ sample in A549 cells and the curve, similar to ours, showed an increase in cytotoxicity between 24 h and 48 h with a shape that is representative of all the samples we looked at [19]. All eight nanoforms also exhibited a dose-dependent increase in ROS as measured using a DCFDA assay (Figure 4). The magnitude of the increase did vary between samples with Mn-03, Mn-04 and Mn-08 producing more ROS than other samples. The measured ROS production peaked at around 50–100 µg/mL, above which concentration the values dropped off again. This is likely because of the decrease in the number of viable cells at these concentrations, although interference from the NPs could also be affecting the readout of this fluorescence-based assay. We have frequently seen that at high concentrations of NPs, particles can settle and deposit on the bottom of the wells and thus affect the absorption of incoming and detection of emitted light.

### 3.4. HepG2 Liver Cells

**Figure 5:**
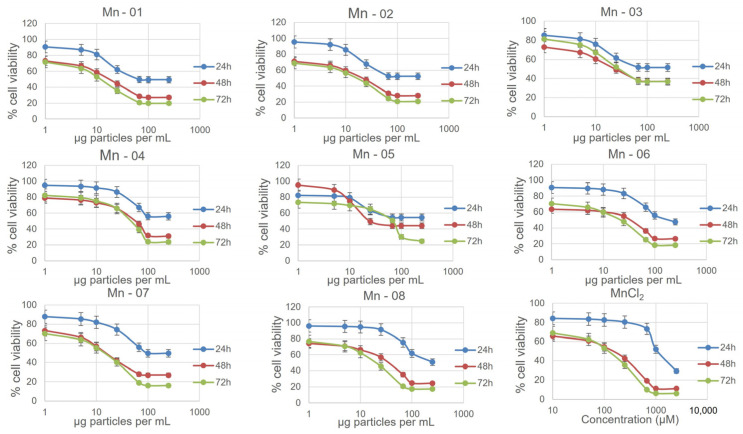
Nanoparticle cytotoxicity in the HepG2 cells as measured using an MTT assay at 24, 38 and 72 h.

**Figure 6:**
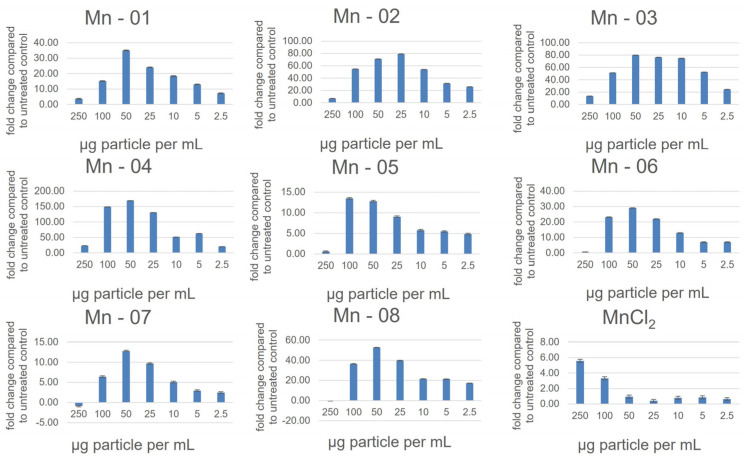
Reactive oxygen species as measured using a DCFDA assay in the HepG2 cells after 24 h exposure.

In the HepG2 cells (Figure 5), NP cytotoxicity closely mirrored what was observed in A549 cells. One minor difference occurred for Mn-05. In the HepG2 cells, this sample did not show a clear increase in toxicity over time in the MTT assay. From our TEM and DLS measurements in both water and cell culture medium, this sample was not noticeably different in size or stability from other bare surfaced samples. In the neutral red assay, the coated samples again showed less cytotoxicity, although now Mn-05 showed the least cytotoxicity of all samples. Mn-05 behaved differently than other samples in this cell line in a manner that is unique to HepG2 cells. In this cell line, the cytotoxicity curves from the neutral red assay more closely aligned with those from the MTT assay compared to what was observed in A549 cells (Figure S4). There was again a dose-dependent response in the DCFDA assay, showing an increase in ROS up to approximately 50 µg/mL before the interferences and cell death caused the values to decrease again (Figure 6). In the HepG2 cells, the higher values for fold change compared to the other two cell lines reflected the very small changes over 24 h in the untreated control. This small control signal also makes it difficult to compare the samples as small changes in the control signal greatly change the fold change. When looking at just the raw signal intensity from the readouts (not normalized to the control), Mn-01, Mn06, Mn-07 and Mn-08 showed the highest production of ROS.

### 3.5. J774A.1 Macrophage Cells

**Figure 7:**
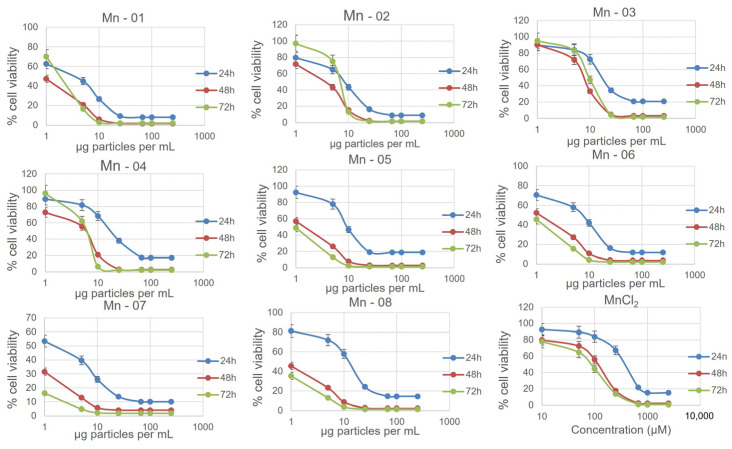
Nanoparticle cytotoxicity in the J774A.1 cells as measured using an MTT assay at 24, 38 and 72 h.

**Figure 8:**
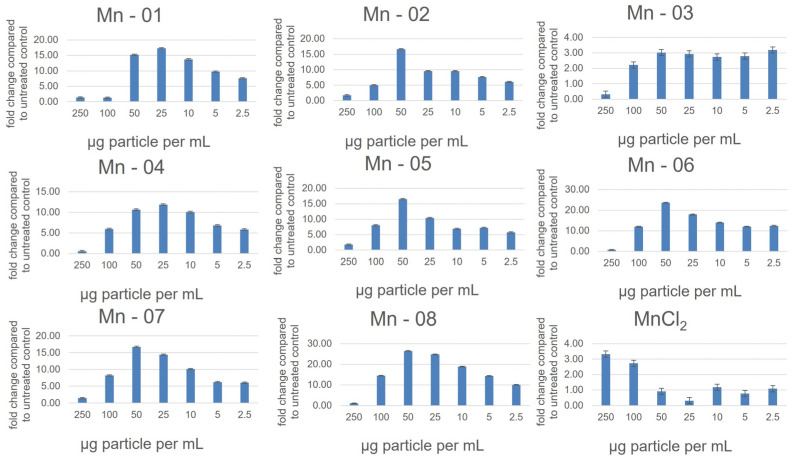
Reactive oxygen species as measured using a DCFDA assay in the J774a.1 cells after 24 h of exposure.

The J774A.1 cells showed the highest levels of cytotoxicity of the three cell lines (Figure 7). As with the A549 and HepG2 cells, the three coated samples, Mn-02, Mn-03 and Mn-04, showed lower cytotoxicity compared to the bare particles in both the MTT and neutral red assays. In the DCFDA assay, all samples showed a dose-dependent increase in ROS except for Mn-03 (Figure 8). Interestingly, this sample also exhibited the lowest cytotoxicity. In drawing conclusions from this sample though, it must be considered that this stearic-acid-coated material was very poorly dispersed in the cell culture medium and thus the quality of the dispersion could impact the biological results. While that could account for lower cytotoxicity, this same dispersion was used in the other two cell lines where dose-dependent increases in ROS were observed, suggesting that this result is unique to this cell line and particle combination.

It has previously been reported that Mn_2_O_3_ particles increase caspase 3 activity but not caspase 1, suggesting that they interact somewhere along the inflammatory signaling pathway. We measured the effect of all eight nanoforms at 10 µg/mL (above this concentration effects were reduced due to cytotoxicity of the particles) on caspase 1 and 3 and found that all eight particles indeed activated caspase 3 but not caspase 1 (Table 5). In fact, for many samples, caspase 1 activity was reduced compared to an untreated control. This suggests that the effect on caspase 3 is universal for Mn_2_O_3_ particles and not unique to any specific nanoform. The magnitude of the increase in caspase 3 activity, however, was not uniform for all particles, with Mn-06 showing a much weaker effect than other particles and Mn-4 exhibiting the highest effect. The sample with the lowest caspase 1 activity was Mn-01.

Comparing the cytotoxicity data from the three cell lines, there was a consistent trend in the cytotoxicity increasing between 24 and 48 h. While there was a dose-dependent increase in ROS production in all three cell lines, these two assays did not show a positive correlation. There is evidence that the production of ROS can have both beneficial and detrimental effects at different concentrations, highlighting how each individual data point represents its own unique microsystem [8]. For J774A.1 cells, Mn-01 and Mn-07 were the most cytotoxic at 24 h; however, Mn-04 and Mn-06 produced higher levels of ROS. Yet, Mn-03 produced significantly less ROS and had lower cytotoxicity, at least in the neutral red assay. For HepG2, Mn-01 and Mn-07 were also more cytotoxic; however, in A549 cells, all samples behaved more similarly to one another. The lack of variation is perhaps not surprising as our physical measurements of size and surface area also showed less difference between the materials than was reported by the producers. Further studies with materials that have larger size differences may be able to elucidate a trend; however, there are relatively few suppliers of materials and individual suppliers do not typically produce materials of more than one size to compare with one another.

## 4. Conclusions

Mn_2_O_3_ is used in a number of commercial applications and there exists a need to understand if there are safety concerns related to human exposure. From the studies reported here, the commercially available materials did not differ significantly in size and shape, and the dispersions of the materials were heterogeneous and highly agglomerated. Mammalian cell exposure highlights a dose-dependent increase in cytotoxicity which typically increases up to 48 h and correlates with the production of reactive oxygen species. The nanoparticles also all activated caspase 3 and not caspase 1 as previously reported [9,10]; however, this activity varied significantly between the different materials and was not obviously correlated with any of the physical or biological measurements. This work reinforces previous conclusions on the importance of using well-characterized nanomaterials for biological studies that are aimed at assessing their potential safety concerns and that will enable read-across to facilitate comparison between similar materials.

## Figures and Tables

**Table 1:** Sample codes, suppliers, surface coatings and nominal sizes for the Mn_2_O_3_ NPs used in this study. Specific surface areas measured by BET are listed along with the values provided by the supplier.

Sample	Supplier	Surface Coating	Size, nm	Specific Surface Area, m^2^/g	
				BET	Supplier ^1^
Mn-01	US Research Nanomaterials	uncoated	30	30.6	150
Mn-02	US Research Nanomaterials	PVP	30	19.6	- ^2^
Mn-03	US Research Nanomaterials	Stearic acid	30	9.3	- ^2^
Mn-04	US Research Nanomaterials	silane	30	36	- ^2^
Mn-05	US Research Nanomaterials	uncoated	<100	40.0	68
Mn-06	mKNano	uncoated	50	28.9	
Mn-07	American Elements	uncoated	<100	29.4	20–50
Mn-08	Nanografi	uncoated	28	30.7	>155

^1^ Supplier data is not available for all metal oxides. ^2^ May be assumed to be similar to the uncoated sample (Mn-01).

**Table 2:** Particle shape and size distribution measured by TEM. Particle size distributions are characterized by the equivalent circular diameter, d_eq_ and aspect ratio with standard deviations (SD) as a measure of the distribution breadth. The number of particles analyzed (n) is provided and the TEM data are compared to size and shape data provided by the supplier.

Sample	TEM						Supplier ^1^	
	Shape ^2^	n ^3^	d_eq_ (Std Error), nm	SD, nm	Aspect Ratio	SD	Shape	Size, nm
Mn-01	SP, EL, SQ	70 ^3^	53 (2)	17	1.3	0.2	rods, flakes	30
Mn-02	SP, EL, IRR	111	54 (2)	17	1.3	0.2		30
Mn-03	SP, EL, IRR	113	56 (3)	27	1.3	0.2		30
Mn-04	SP, EL, IRR, rods, plate	130	49 (2)	21	1.3	0.2		30
Mn-05	SP, EL, rods	163	47 (2)	13	1.3	0.2	spherical, rods	<100, aver 35
Mn-06	SP, EL, rods, plates	83	41 (2)	17	1.3	0.2		50
Mn-07	SP, EL, rods, plates	81	37 (2)	14	1.3	0.2		<100
Mn-08	SP, EL, IRR	84	37 (2)	15	1.5	0.3	spherical	28

^1^ Size and shape are not provided for all samples by the supplier. ^2^ Shapes are abbreviated as SP = (nearly) spherical; EL = ellipsoidal or oval; IRR = irregular, typically an irregular polygon; SQ = square. Rods are high-aspect-ratio particles that are typically overlapped with other particles and therefore not analyzed. ^3^ Fewer than 100 particles were analyzed for samples that were more heavily agglomerated.

**Table 3:** Dynamic light scattering data (Z-average and PDI) and zeta potential measurements for aqueous dispersions and Mn content as determined by ICP-MS for Mn_2_O_3_ NPs. The standard deviation from three measurements is given in brackets.

Sample	Z-Average, nm	PDI	Zeta Potential, mV	Mn Content (%) (ICP-MS)
Mn-01	212 (5)	0.34 (0.05)	28.5 (0.2)	63 (1)
Mn-02	266 (9)	0.36 (0.03)	−11.2 (0.3)	62 (2)
Mn-03	>1000	>0.4	2.1 (0.2)	61 (1)
Mn-04	202 (5)	0.27 (0.03)	32.5 (0.6)	63 (2)
Mn-05	229 (6)	0.24 (0.02)	33.2 (0.8)	65 (1)
Mn-06	233 (7)	0.33 (0.04)	29.1 (0.4)	63 (1)
Mn-07	244 (8)	0.36 (0.06)	28.3 (0.9)	63 (2)
Mn-08	211 (5)	0.29 (0.05)	24.7 (0.3)	64 (2)

**Table 4:** Dynamic light scattering data (Z-average, nm and PDI) for suspensions of Mn_2_O_3_ NPs in cell culture medium over a period of 72 h. The standard deviation from three measurements is given in brackets.

Sample	0 h		24 h		48 h		72 h	
	Z-Average	PDI	Z-Average	PDI	Z-Average	PDI	Z-Average	PDI
Mn-01	225 (6)	0.19 (0.03)	215 (7)	0.16 (0.04)	223 (3)	0.16 (0.03)	234 (4)	0.22 (0.03)
Mn-02	247 (9)	0.23 (0.01)	244 (8)	0.26 (0.03)	253 (5)	0.26 (0.04)	255 (9)	0.28 (0.04)
Mn-03	>1000	>0.5	>1000	>0.5	>1000	>0.5	>1000	>0.5
Mn-04	258 (8)	0.25 (0.05)	252 (9)	0.24 (0.04)	247 (7)	0.21 (0.03)	253 (9)	0.23 (0.03)
Mn-05	236 (6)	0.23 (0.03)	235 (3)	0.19 (0.02)	235 (3)	0.21 (0.02)	247 (7)	0.26 (0.03)
Mn-06	244 (9)	0.21 (0.01)	237 (6)	0.22 (0.01)	247 (8)	0.24 (0.03)	247 (5)	0.22 (0.03)
Mn-07	237 (5)	0.23 (0.01)	243 (2)	0.22 (0.01)	238 (5)	0.21 (0.01)	239 (6)	0.22 (0.03)
Mn-08	218 (5)	0.24 (0.04)	234 (4)	0.30 (0.05)	234 (3)	0.26 (0.04)	228 (6)	0.24 (0.03)

**Table 5:** Caspase 1 and caspase 3 activity after incubation with NPs for 24 h in J774A.1 cells. Values are reported as a percentage of activity compared to that measured in an untreated control sample. Caspase activities are reported at 10 µg/mL NPs.

Sample ID	Caspase 1 Activity versus Control (±3%)	Caspase 3 Activity versus Control (±3%)
Mn-01	76%	170%
Mn-02	100%	170%
Mn-03	82%	142%
Mn-04	82%	200%
Mn-05	100%	149%
Mn-06	84%	110%
Mn-07	100%	158%
Mn-08	100%	152%

## Data Availability

Data are contained within the article and supplementary materials.

## Supplementary Materials

The following supporting information can be downloaded at: https://www.mdpi.com/article/10.3390/nano14020198/s1, Figure S1: Additional TEM images; Figure S2: Histograms for equivalent circular diameter; Figure S3: Histograms for equivalent circular diameter; Figure S4: Neutral red assay.
