# Supplementary material for: Characterization and Cellular Toxicity Studies of Commercial Manganese Oxide Nanoparticles
Source: Nanomaterials (Basel). 2024 Jan 16;14(2):198. doi: 10.3390/nano14020198 (PMC10821457; doi:10.3390/nano14020198)
Supplement: Supplementary file 1 [file nanomaterials-14-00198-s001.zip › nanomaterials-2746821-supplementary.pdf]

# Characterization and Cellular Toxicity Studies of Commercial Manganese Oxide Nanoparticles

**Linda J. Johnston,[1] Xiaomei Du,[2] Andre Zborowski,[2] and David C. Kennedy[1,*]**

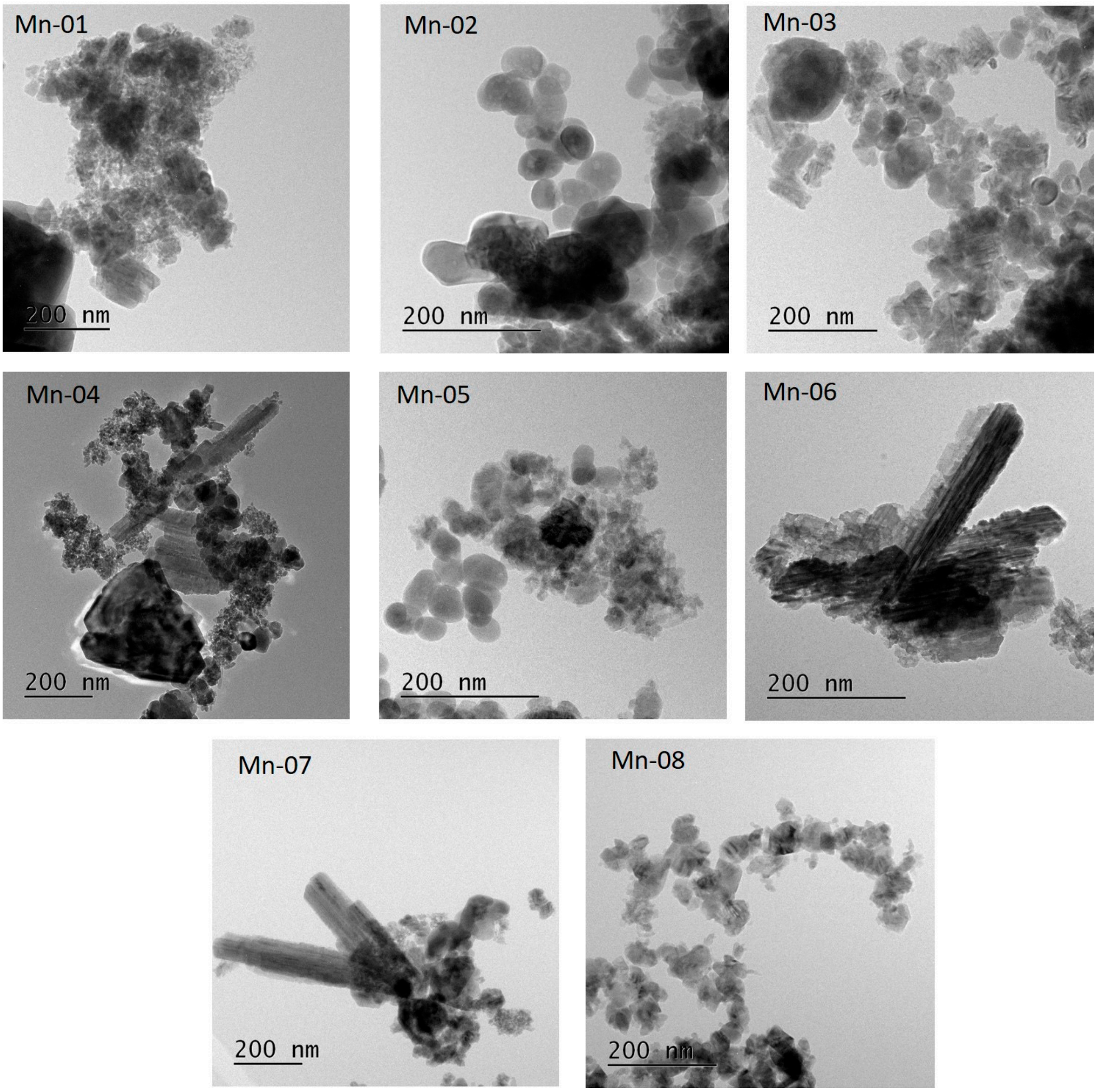


**Figure S1**. Additional TEM images for $Mn_2O_3$ nanoparticles.

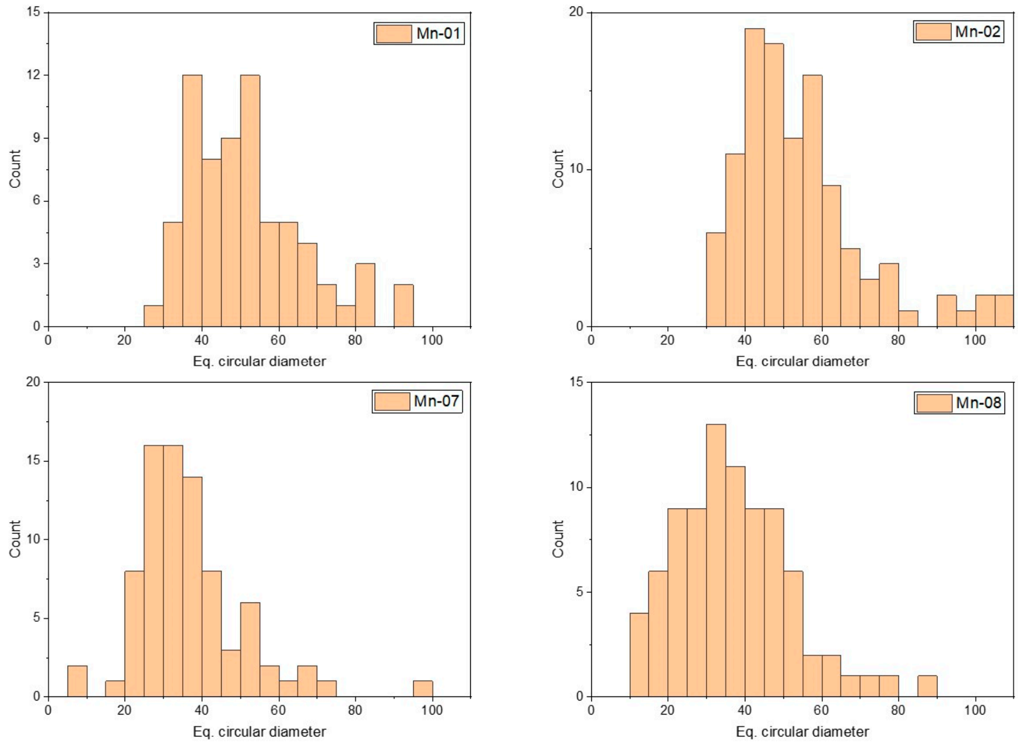


**Figure S2**. Histograms for equivalent circular diameter for $Mn_2O_3$ nanoparticles.

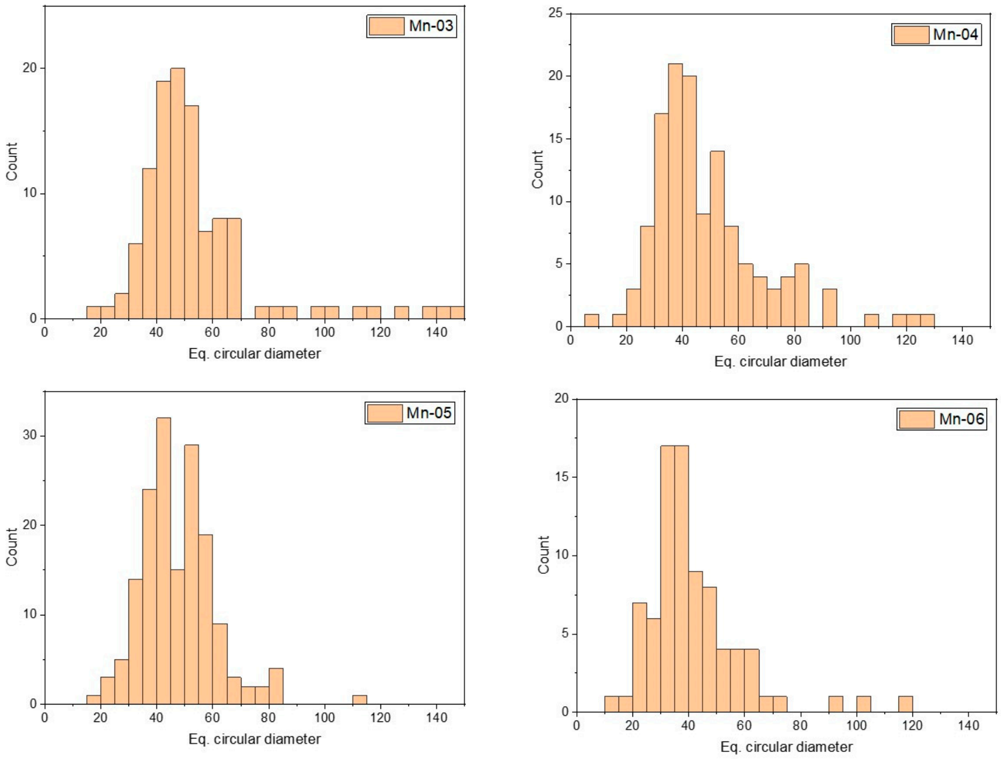


**Figure S3**. Histograms for equivalent circular diameter for $Mn_2O_3$ nanoparticles.

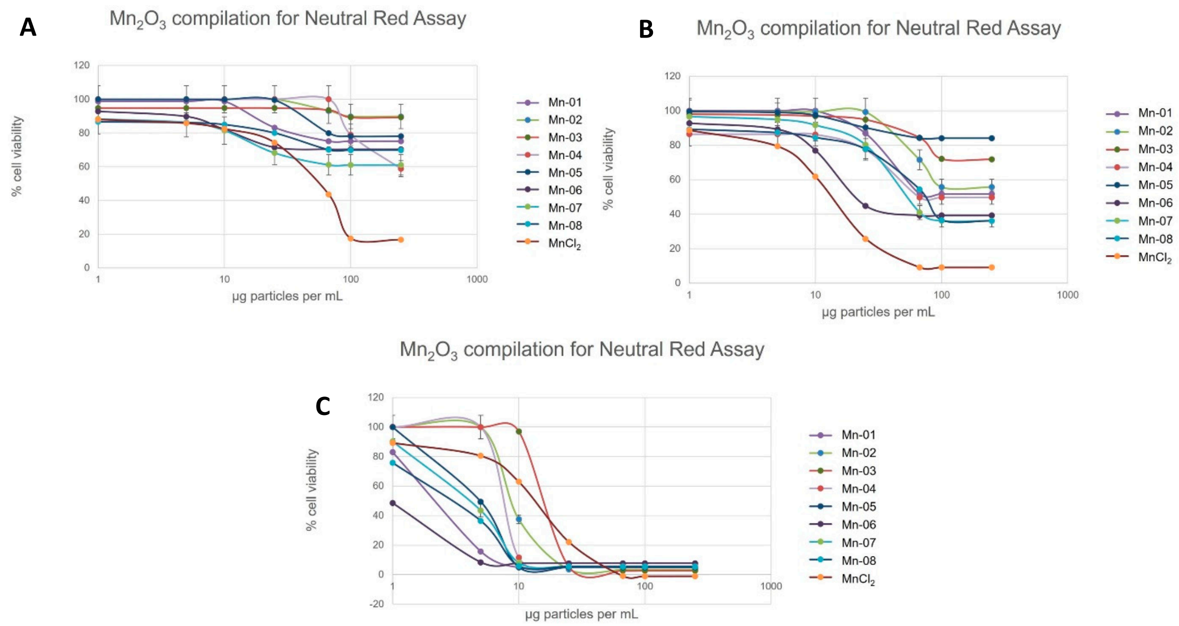


**Figure S4**. Neutral red assay results at 72 h for $Mn_2O_3$ treated cells from 1-250 µg/mL in A549 (A), HepG2 (B) and J774a.1 (C) cells.
